# Supplementary material for: The phenotypic spectrum of terminal 6q deletions based on a large cohort derived from social media and literature: a prominent role for DLL1
Source: Orphanet J Rare Dis. 2023 Mar 19;18:59. doi: 10.1186/s13023-023-02658-w (PMC10024851; doi:10.1186/s13023-023-02658-w)
Supplement: Supplementary file 2 — Additional file2. Figure S1: Case collection from the Chromosome 6 Project and literature. Figure S2: Developmental delay in children older than 2 years of age. Figure S3: Delayed Myelination. Figure S4: Dental problems. Table S2: HI and pLI scores. Table S4: Genes mentioned in the Discussion and the clinical characteristics to which they may contribute. [file 13023_2023_2658_MOESM2_ESM.pdf]

Additional File 2: Supplementary files

The phenotypic spectrum of terminal 6q deletions based on a large cohort derived from social media and literature: a prominent role for *DLL1*

Aafke Engwerda, Wilhelmina S. Kerstjens-Frederikse, Nicole Corsten-Janssen, Trijnie Dijkhuizen and Conny M. A. van Ravenswaaij-Arts

Supplementary Figures

Figure S1. Case collection from the Chromosome 6 Project and literature
Figure S2. Developmental delay in children older than 2 years of age
Figure S3. Delayed Myelination
Figure S4. Dental problems

Supplementary Table

Table S2. HI and pLI scores
Table S4. Genes mentioned in the Discussion and the clinical characteristics to which they may contribute

Figure S1. Case collection from the Chromosome 6 Project and literature

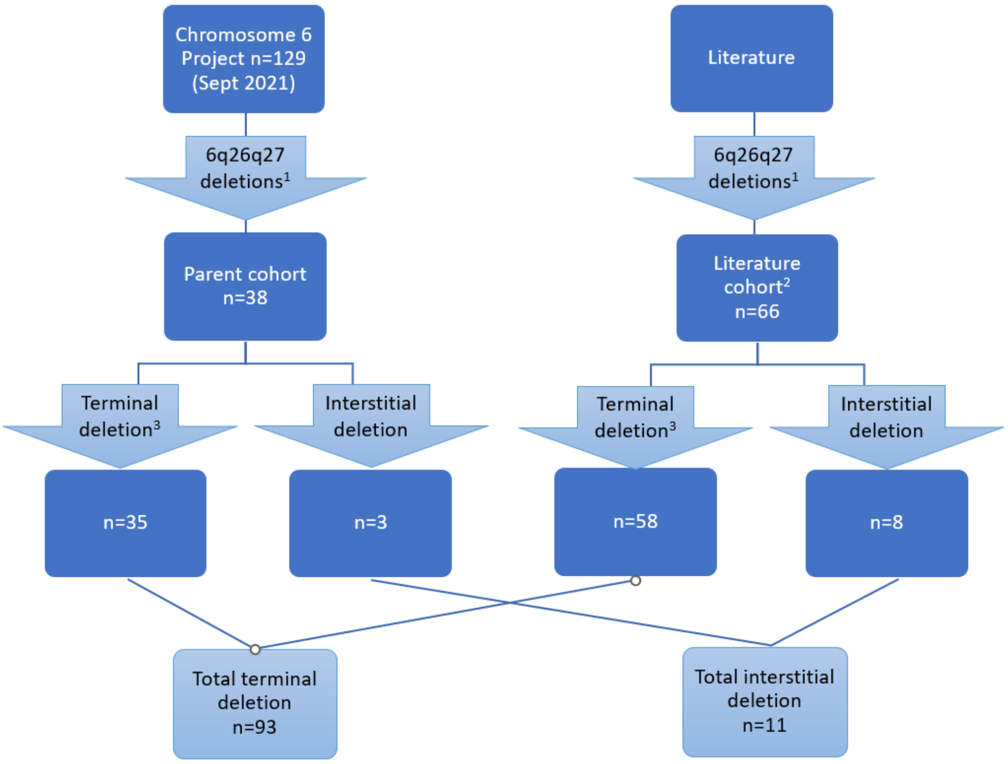

The number of terminal and interstitial 6q deletion cases derived from the Chromosome 6 Project and the literature.

[1] Defined as a deletion within the 6q26q27 region (161,000,001–171,115,067).

[2] Case reports involving terminal 6q deletions collected using PubMed with the search criteria (deletion or monosomy) and (6q26 or 6q27 or terminal 6q). References were checked for additional relevant case reports. Publications reporting detailed clinical information and microarray results or comparably detailed breakpoint analyses were included.

[3] Defined as a deletion including the most distally located gene (*PDCD2,* MIM*600866).

Figure S2. Developmental delay in children older than 2 years of age

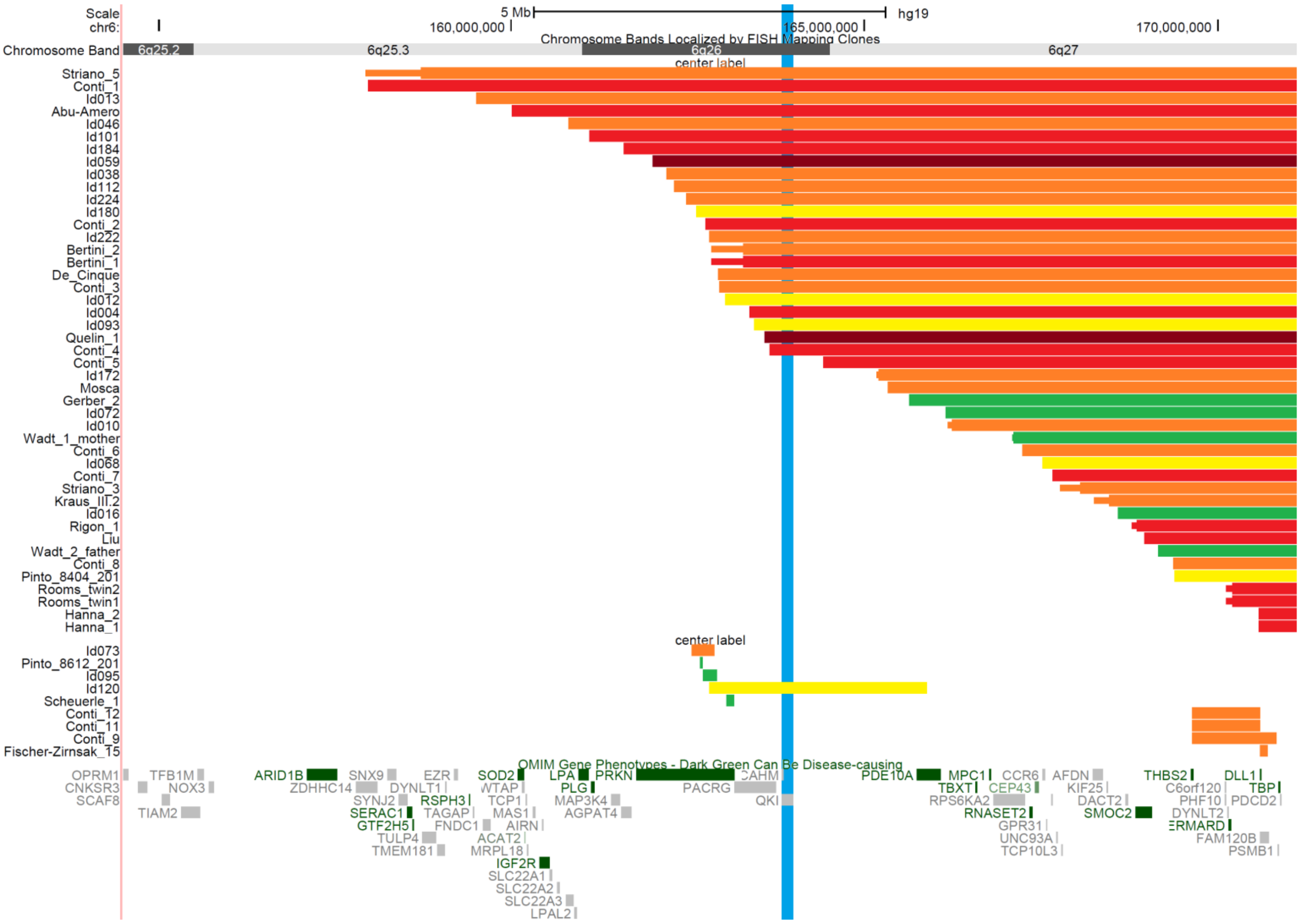

The deletion in each patient for whom development could be categorised is represented by a horizontal bar. Development is categorised as normal (IQ >85, green bar), borderline (IQ 70-85, yellow), mild (IQ 50-70, orange), moderate (IQ 30-50, red) or severe (IQ <30, dark red) delay. The gene *QKI* is represented by a vertical blue bar (see manuscript Discussion).

Figure S3. Delayed Myelination

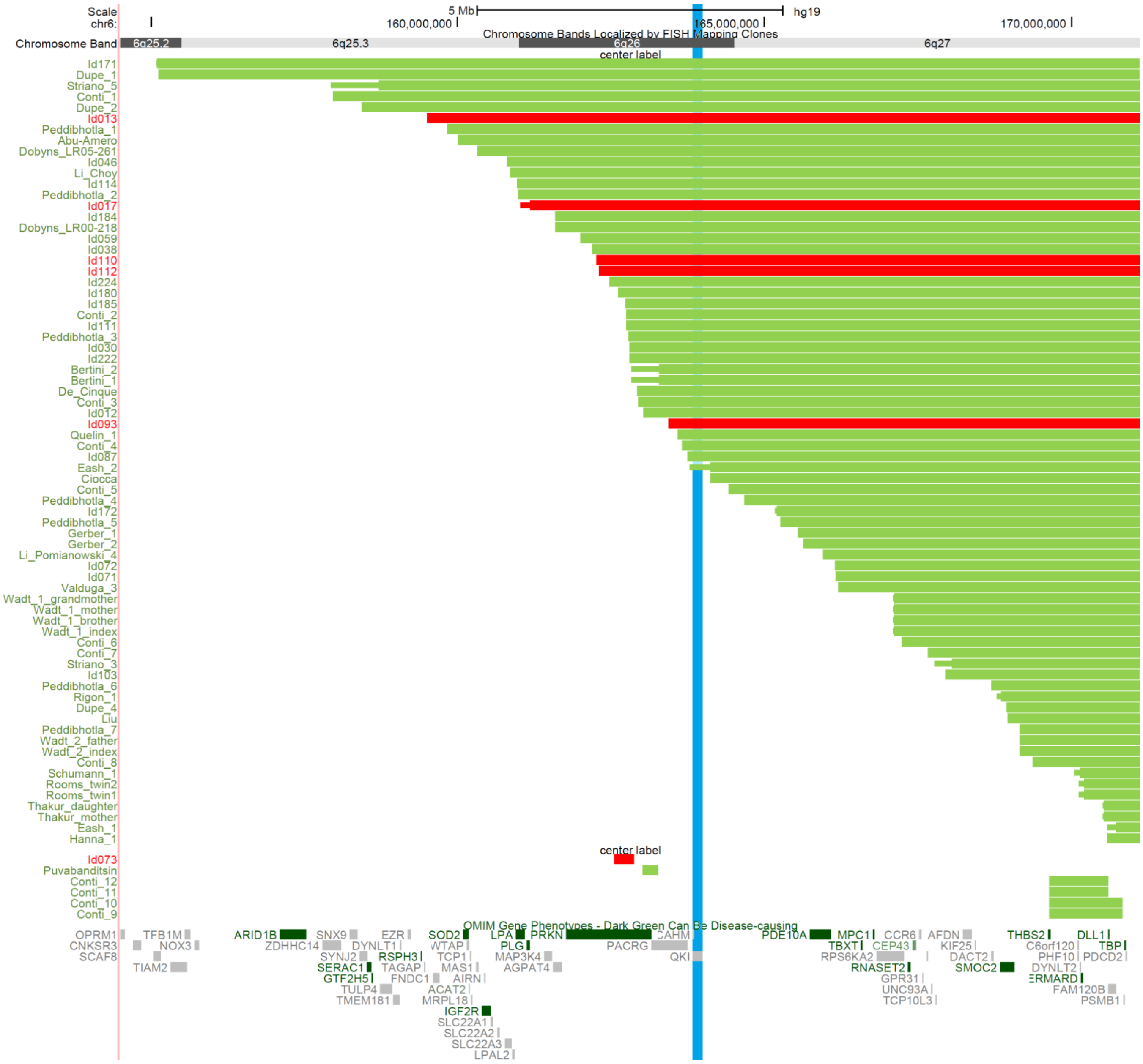

The deletion in each patient for whom delayed myelination was present (red) or absent (green) is represented by a horizontal bar. The gene *QKI* is represented by a vertical blue bar (see manuscript Discussion).

Figure S4. Dental problems

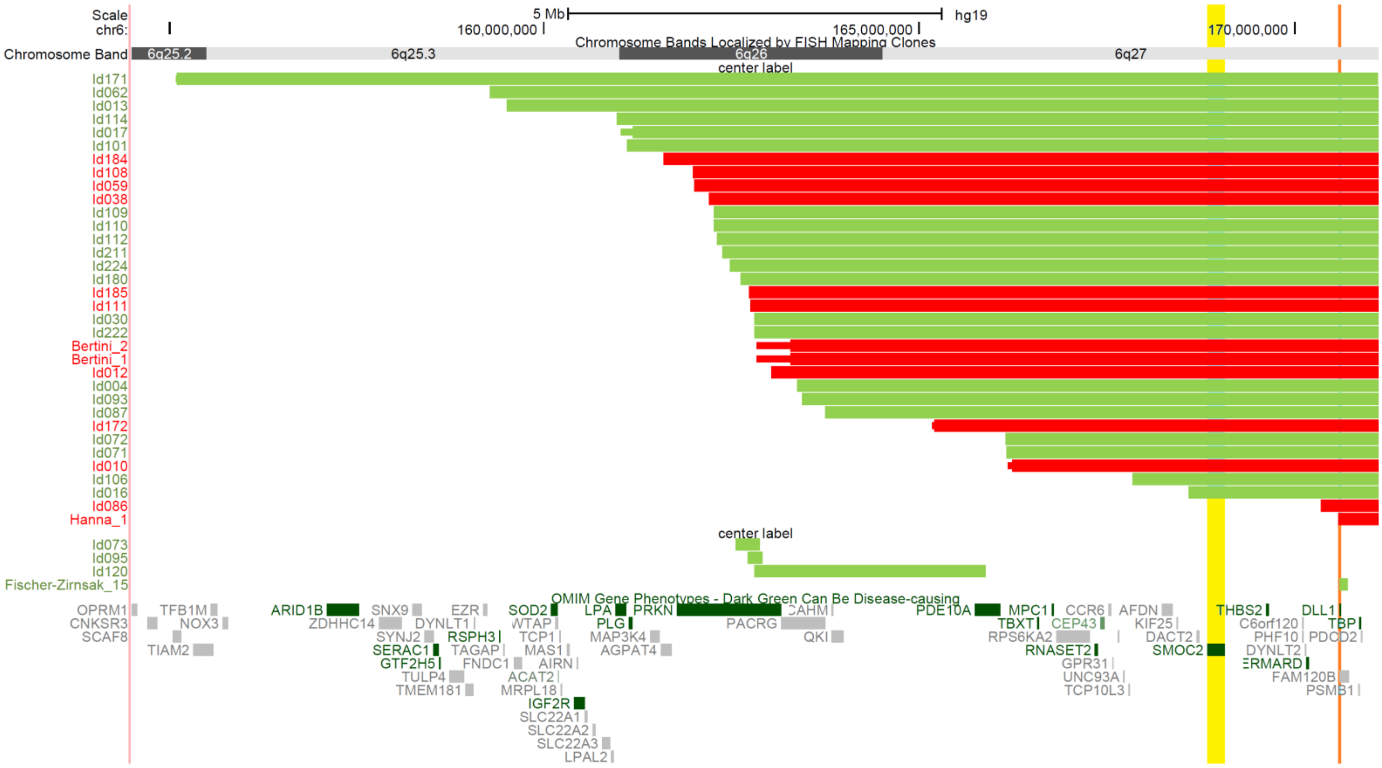

The deletion in each patient for whom dental problems were present (red) or absent (green) is represented by a horizontal bar. The gene *SMOC2* is represented by a vertical yellow bar and the gene *DLL1* is represented by a vertical orange bar. *SMOC2* is related to dental abnormalities in pathogenic homozygous variants (see manuscript Results and Discussion).

Table S2. HI and pLI scores

Location	Gene	MIM*	% HI	pLI
6q27	*PDCD2*	600866	69.48	0.00
	TBP	600075	6.48	0.02
	PSMB1	602017	33.38	0.97
	FAM120B	612266	80.03	0.00
	DLL1	606582	4.65	1.00
	ERMARD (C6orf70)	615532	84.86	0.00
	DYNLT2	186977	-	0.00
	PHF10	613069	18.13	0.00
	C6orf120	616987	92.18	-
	THBS2	188061	59.96	0.56
	SMOC2	607223	55.41	0.00
	DACT2	608966	93.50	0.01
	KIF25	603815	95.70	0.00
	AFDN	159559	-	1.00
	TCP10L3	187020	-	0.00
	UNC93A	607995	85.97	0.00
	GPR31	602043	91.50	0.02
	CCR6	601835	84.13	0.01
	CEP43	605392	-	0.00
	RNASET2	612944	88.27	0.00
	RPS6KA2	601685	64.44	0.02
	MPC1	614738	49.44	0.04
	TBXT	601397	-	0.06
	PDE10A	610652	52.14	1.00
6q26	***QKI***	609590	4.10	0.75
	CAHM	615930	-	-
	PACRG	608427	10.61	0.00
	PRKN	602544	0.77	0.00
	AGPAT4	614795	61.98	0.10
	MAP3K4	602425	59.19	1.00
	PLG	173350	40.11	0.01
	LPA	152200	78.22	0.00
6q25.3	*LPAL2*	611682	-	-
	SLC22A3	604842	49.06	0.00
	SLC22A2	602608	63.53	0.00
	SLC22A1	602607	75.70	0.00
	AIRN	604893	-	-
	IGF2R	147280	53.96	1.00
	MAS1	165180	28.09	0.00
	MRPL18	611831	49.78	0.00
	TCP1	186980	5.10	1.00
	ACAT2	100678	53.11	0.00
	WTAP	605442	6.76	1.00
	SOD2	147460	0.99	0.15
	FNDC1	609991	75.28	0.00
	TAGAP	609667	82.32	0.85

	RSPH3	615876	86.62	0.00
	EZR	123900	8.64	0.18
	DYNLT1	601554	66.63	0.00
	TMEM181	613209	67.29	0.01
	GTF2H5	608780	30.21	0.05
	SERAC1	614725	61.19	0.00
	SYNJ2	609410	63.42	0.00
	SNX9	605952	40.36	0.71
	ZDHHC14	619295	29.53	0.72
	ARID1B	614556	14.17	1.00
	NOX3	607105	38.77	0.00
	TFB1M	607033	53.74	0.00
6q25.2	*TIAM2*	604709	53.43	0.68
	SCAF8	616024	25.56	1.00

All the OMIM genes studied, extending from 6q27 to 6q25.2 (16 Mb). Predicted HI-genes are highlighted in bold. HI and pLI scores were derived from https://decipher.sanger.ac.uk in May 2021

Table S4. Genes mentioned in the Discussion and the clinical characteristics to which they may contribute

Gene	May contribute to
DLL1	**small head size, dysplastic outer ears, hypertelorism, vision problems, abnormal eye movements, dental abnormalities, feeding problems, recurrent infections, respiratory problems,** congenital heart defects, **spinal cord abnormalities, abnormal vertebrae, scoliosis, joint hypermobility, brain abnormalities (ventriculomegaly/hydrocephaly, corpus callosum abnormality and cortical dysplasia),** PNH, **seizures, hypotonia, ataxia, torticollis, balance problems, developmental delay, sleeping problems, hyperactivity,** autism spectrum disorder
ERMARD (C6orf70)	PNH
PHF10	structural brain abnormalities
THBS2	congenital heart defects, structural brain abnormalities
SMOC2	dental problems
TBXT	hemivertebrae
QKI	cardiomyopathy, **delayed myelination**, **developmental delay**

PNH = periventricular nodular heterotopia

A clinical characteristic is depicted in bold when haploinsufficiency of the associated gene is expected to be causal for the characteristic.
